# Pott’s Puffy Tumor: A Potentially Deadly Complication of Sinusitis

**DOI:** 10.7759/cureus.6351

**Published:** 2019-12-11

**Authors:** Matthew J Joo, Kurt E Schapira

**Affiliations:** 1 Emergency Medicine, Brooke Army Medical Center, San Antonio, USA

**Keywords:** sinusitis, osteomyelitis, pott puffy tumor

## Abstract

Pott’s puffy tumor (PPT) is a rare but potentially deadly complication of bacterial sinusitis that consists of subperiosteal abscess and osteomyelitis of the frontal bone. It is best diagnosed with computerized tomography (CT) with intravenous contrast or magnetic resonance imaging and treated with early broad-spectrum antibiotics and surgical intervention. We describe a case of PPT in a 7-year-old patient and present the CT images.

## Introduction

Sinusitis is a common condition affecting an estimated one in eight people in the United States every year and usually resolves without treatment [1, 2]. The most common cause is viral infection, however approximately 0.5-2% of these patients will have or develop bacterial sinusitis [3]. While 80% of cases of sinusitis will resolve without antibiotics, significant complications can arise if bacterial sinusitis is left untreated [2]. Pott’s puffy tumor (PPT) is a rare condition that consists of subperiosteal abscess and osteomyelitis of the frontal bone [4]. This disease is a rare complication of bacterial sinusitis, or even more rarely trauma, and most frequently occurs in pediatric and adolescent populations [5]. We describe a case of PPT in a pediatric patient.

## Case presentation

**Figure 1:**
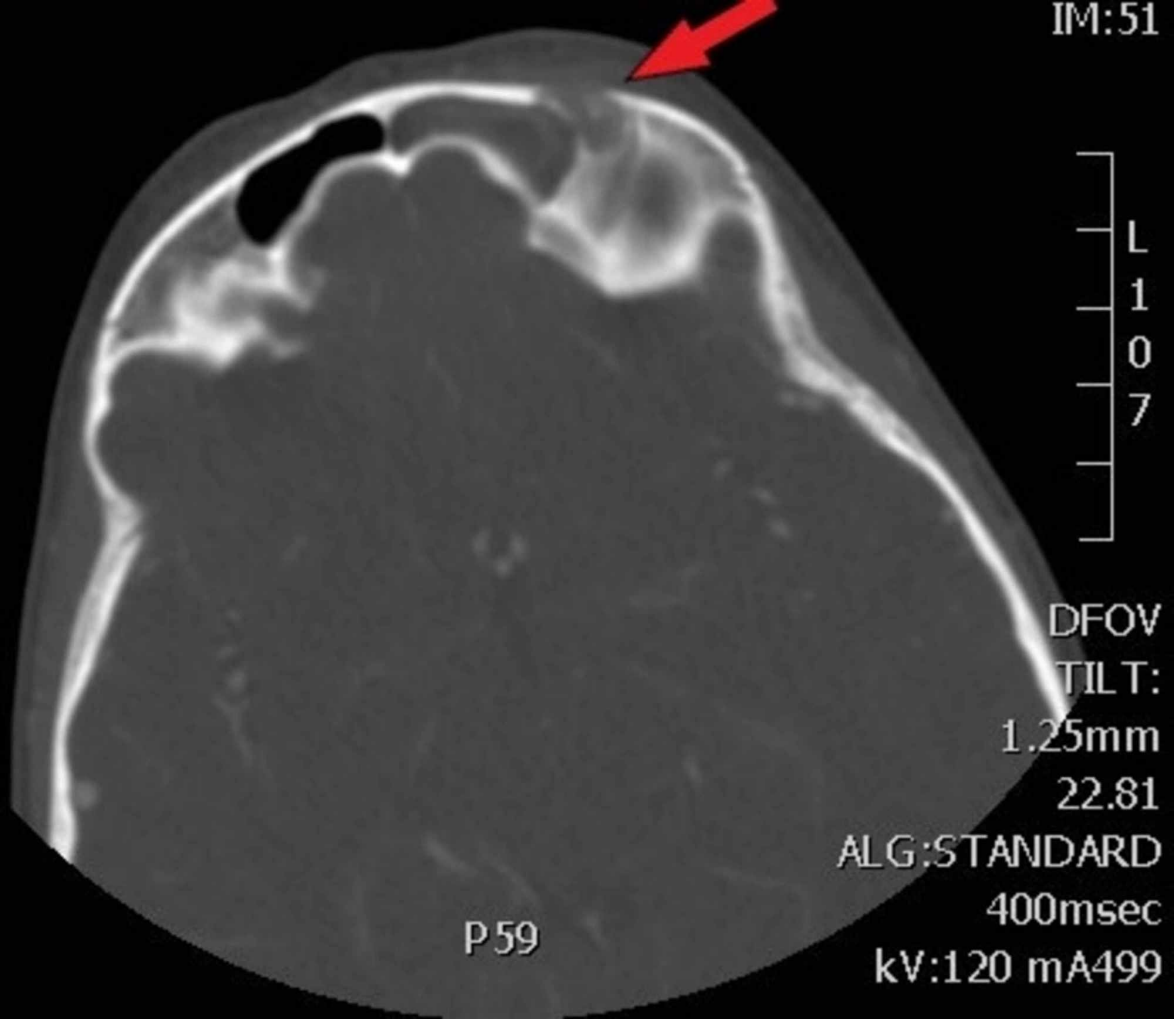
Axial CT scan with arrow indicating erosion of the left frontal bone and opacification of the left frontal sinus

**Figure 2:**
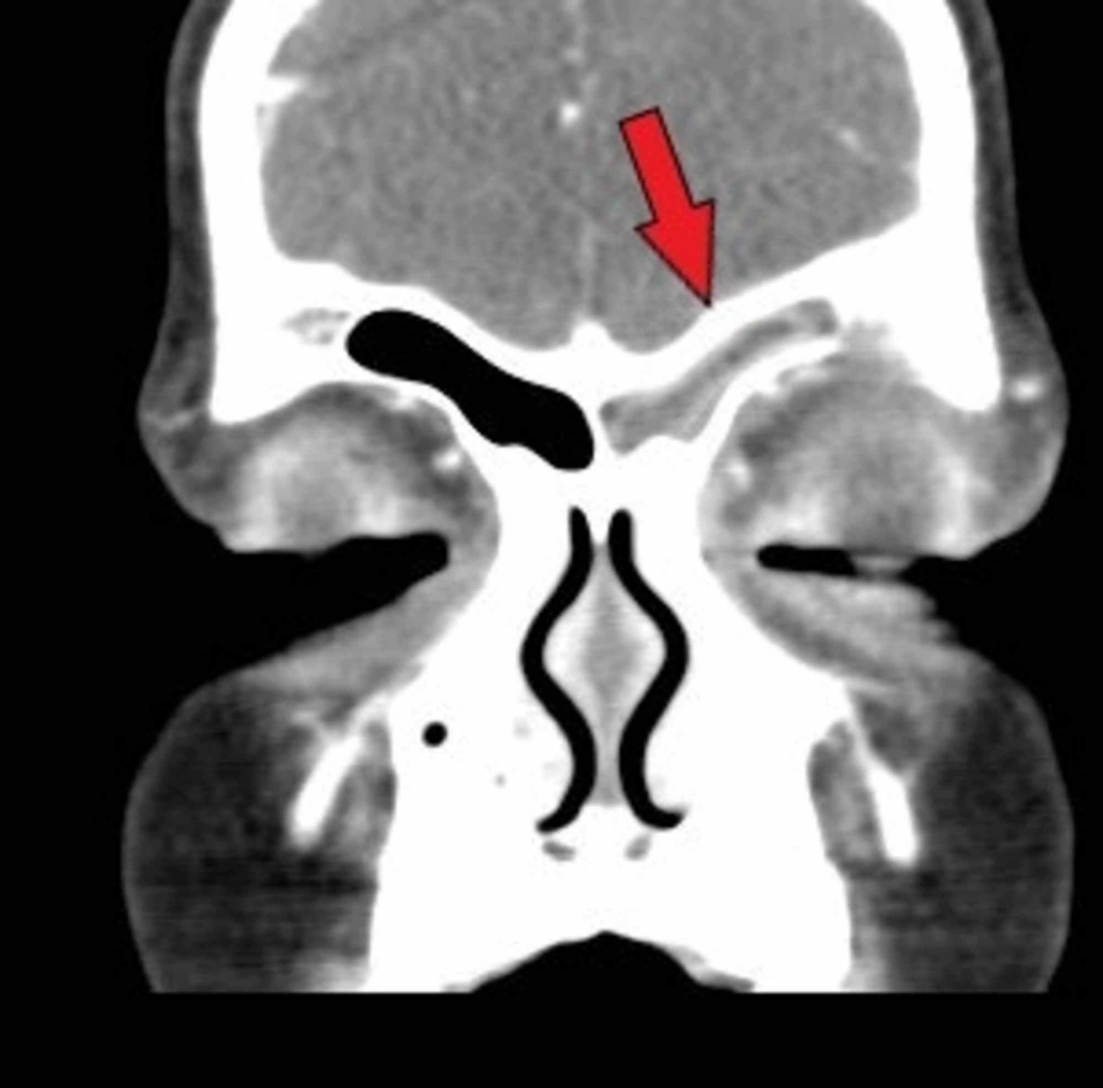
Coronal CT scan with arrow indicating opacification of the left frontal sinus

A 7-year-old female with no significant past medical or surgical history presented to the emergency department (ED) with two weeks of headache and left-sided upper eyelid redness with five days of periorbital swelling. The patient’s parent stated she was initially seen by her pediatrician two weeks ago, diagnosed with allergies, and was prescribed cetirizine with no relief. Five days prior to ED presentation the swelling began and the redness worsened. The patient was taken to an urgent care and given oral diphenhydramine with no relief. The patient’s mother reported she was sent to the ED today by her pediatrician for further evaluation. The patient’s parents denied fever during this two-week course. They reported the patient had some mild congestion and cough. Vitals signs were normal except for a mild tachycardia of 113. The patient was very well appearing with physical exam findings of mild erythema and edema to the left upper periorbital region and eyelid. Cranial nerves were normal as tested. The patient denied pain with extraocular movements. Computerized tomography (CT) with intravenous (IV) contrast was obtained demonstrating acute left frontal sinusitis with associated osteomyelitis eroding the outer table of the left frontal sinus with associated cellulitis of the supraorbital soft tissues and orbital soft tissues above the superior rectus, concerning for Pott’s puffy tumor and periorbital cellulitis (Figures 1, 2). Urgent consultations to otolaryngology and pediatrics were made and the patient was admitted for surgical debridement and biopsy. The patient was admitted and taken directly to the operating room, she was started on IV ampicillin/sulbactam. Her operative course was complicated by a need for two separate surgeries to remove and debride tissue. The patient was discharged home without further complications after eight days, and placed on oral amoxicillin/clavulanate outpatient.

## Discussion

Pott’s puffy tumor is an uncommon condition that involves both osteomyelitis and periosteal abscess of the frontal bone first described by Sir Percival Pott in 1760 [4,6]. The condition is important to diagnose early as complications of the “tumor” can result in meningitis, frontal lobe abscess, cavernous sinus thrombosis, and epidural or subdural abscess [4]. Symptoms can vary from headache, periorbital swelling, swelling of the forehead, to purulent drainage, cutaneous fistulas, altered mental status, and cranial nerve deficits but fever can be absent [4,5,7]. Diagnosis can be made with CT with IV contrast, however magnetic resonance imaging can be useful to characterize the extent of the disease [5]. Management consists of early broad-spectrum IV antibiotics with surgical intervention to drain abscess and debride tissue [4,5,7,8]. Intracranial spread may require neurosurgical intervention [8].

## Conclusions

Pott’s puffy tumor is a rare complication of sinusitis; however, it can be lethal if left undiagnosed. PPT should be considered in any patient with suspected sinusitis as emergent imaging, initiation of antibiotics and surgical consultation are required to prevent morbidity and mortality.
